# Role of noncanonical histone H2A variant, H2A.Z, to maintain proper centromeric transcription and chromosome segregation

**DOI:** 10.1016/j.jbc.2025.108464

**Published:** 2025-03-28

**Authors:** Mahmuda Akter, Xiaoai Lyu, Jiaxing Lu, Xiao Wang, Tyson Phonesavanh, Hao Wang, Hongtao Yu, Jungseog Kang

**Affiliations:** 1 Arts and Science, New York University at Shanghai, Shanghai, China; 2 School of Life Sciences, Westlake University, Hangzhou, Zhejiang, China Westlake Laboratory of Life Sciences and Biomedicine, Hangzhou, Zhejiang, China; 3 NYU-ECNU Center for Computational Chemistry at NYU Shanghai, Shanghai, China

**Keywords:** chromosome segregation, centromeric transcription, histone variants, mitotic kinases, H2A.Z

## Abstract

The genome stability of eukaryotic cells is ensured by proper regulation of histones and their variants. H2A.Z, a conserved and essential histone H2A variant, plays a crucial role in this process by regulating various chromatin-related processes such as gene expression, heterochromatin formation, DNA damage repair, and chromosome segregation. It has two isoforms, H2A.Z1 and H2A.Z2, also known as H2AFZ and H2AFV, respectively, which perform both redundant and nonredundant roles in maintaining genome stability. In this study, we investigated the isoform-specific mitotic functions of H2A.Z in HeLa cells. Our studies revealed that the depletion of H2AFV or H2AFZ did not alter the overall cell cycle profile. However, H2AFV depletion significantly increased the formation of micronuclei, indicating defects in chromosome segregation. Additionally, H2AFV depletion led to the accumulation of DNA damage at various nuclear loci including centromeres. Interestingly, we discovered that H2AFV depletion significantly increased centromeric transcription, which may interfere with proper centromere function. Furthermore, we discovered that a mitotic kinase, Aurora B, binds to both H2AFV and H2AFZ, but preferentially to H2AFV. Inhibition of Aurora B activity by hesperadin disrupted proper centromeric transcription but not significantly centromeric localization of H2A.Z. Collectively, these data demonstrated that the H2A.Z isoforms play distinctive regulatory roles in maintaining proper centromeric transcription and DNA repair, ensuring accurate chromosome segregation.

Genome stability is essential for proper functioning of eukaryotic cells, and its dysregulation results in various diseases in humans. Chromatin, the carrier of genetic information, was dynamically regulated by histone octamers consisting of H2A, H2B, H3, and H4 to keep genome stability. They control chromatin accessibility, compactness, and effector recruitment through various posttranslational modifications. Posttranslational modification like methylation, acetylation, ubiquitination, sumoylation, or phosphorylation mostly occurs at the lysine or serine/threonine residues of histone tails and provides specific codes for recruitment of downstream effectors (1). Furthermore, histone variants replacing the canonical histones provide additional fine tuning of specific functions during cell cycle progression (2, 3).

H2A variants include H2A.B, H2A.X, H2A.Z, and macroH2A (4). Among them, H2A.Z is highly conserved from yeast to human and plays a fundamental role in genetic stability of eukaryotic cells. It controls gene expression, replication initiation, heterochromatin formation, DNA damage repair, and chromosome segregation, and its dysregulation was shown to facilitate tumorigenesis (5, 6, 7). It is abundantly expressed, up to 5% of the total level of the H2A (8), and incorporated into chromosomes for its various functions throughout the cell cycle. It has two isoforms, H2A.Z1 and H2A.Z2.1, also known as H2AFZ and H2AFV, respectively, and one splicing variant, H2A.Z2.2. H2AFZ and H2AFV are encoded in different regions of chromosomes but their protein sequences are exactly same except three amino acids. Interestingly, they perform both redundant and nonredundant functions in regulating chromatin dynamics *via* specific binding to their effectors (9, 10). For instance, a recent study showed that H2AFZ controls cell cycle progression from G1 to S phase through c-Myc regulation but H2AFV controls mitotic progression through chromatid cohesion regulation (11). Their localization is also tightly regulated for their specific functions. H2A.Z is enriched in transcription start sites and enhancer DNA regions to regulate gene expression (5, 10). It is also shown to localize at the pericentromeric heterochromatin and recruit HP1 for establishing a high-order chromatin structure (12, 13, 14). Interestingly, H2AFV is recruited to the DNA damage sites and its dynamic exchange is promoted by sumoylation (15, 16, 17). Furthermore, H2AFV, most likely at the centromere, regulates Sgo1, chromosome passenger complex (CPC), or CENP-A/C localization, so that its inhibition results in gross defects in chromosome segregation such as premature sister chromatid separation and micronuclei (11, 14). But how H2A.Z isoforms differentially regulate centromeric chromatin dynamics for accurate chromosome segregation is not clear.

Centromere is composed of pericentromeric and centromeric chromatin (18). Pericentromeric chromatin displays heterochromatin signatures such as H3K9 trimethylation and H4K20 trimethylation (19, 20). However, a significant amount of noncoding RNA transcription has been detected throughout centromeres, and the centromeric transcription was shown to play an important role in chromosome segregation fidelity (21, 22, 23). Centromeric localization of CENP-A/C is dependent on centromeric RNAs. Sister chromatid cohesion is strengthened by ongoing centromeric RNA transcription. Interestingly, centromeric transcription starting from pericentric chromatin is negatively regulated by H2A.Z ortholog in yeast (24, 25). Consistently, H2A.Z was shown to localize at the pericentromeric and centromeric chromatin and recruit HP1 for heterochromatin formation in mammals (12, 13, 26, 27). The important role of H2A.Z isoforms in transcription regulation has been studied in detail but their distinctive roles in centromeric transcription in mammals have not been studied carefully.

Here, we have studied how H2A.Z isoforms differentially regulates chromosome segregation process in human cells. H2AFV isoform has an important regulatory role in chromosome segregation such that its depletion results in increased micronuclei and spindle assembly checkpoint (SAC) maintenance defects. Furthermore, we showed that a mitotic kinase, Aurora B, binds to both H2AFV and H2AFZ, but preferentially to H2AFV. Inhibition of Aurora B notably deregulates centromeric RNA transcription but not significantly H2A.Z level at the centromeric chromatin. Therefore, our studies reveal isoform-specific nonredundant functions of H2A.Z in chromosome segregation and genome stability.

## Results

### Two isoforms of H2A.Z display nonredundant functions in mitotic processes

**Figure 1:**
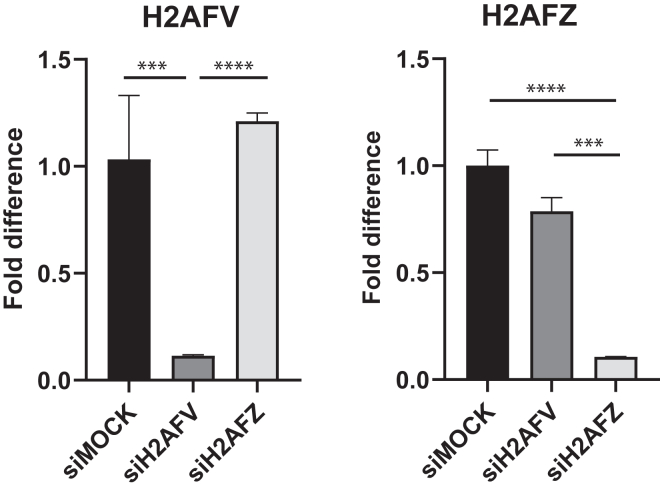
**Isoform-specific depletion by H2A.Z RNAi**. mRNA levels of RNAi cells. HeLa Tet-on cells were transfected by siRNA oligos targeting H2AFV or H2AFZ for 48 h. cDNAs were synthesized from total RNAs of the cells and used for qPCR to examine their mRNA levels. Mean value of mRNA levels (three technical replicates) was shown with standard deviation. Two biological replicates showed a similar trend. Two-tailed *p* value was calculated by unpaired student *t* test. ∗∗∗∗ means *p* value is less than 0.0001, ∗∗∗ less than 0.001, and ∗∗ less than 0.01. NS stands for not significant. cDNA, complementary DNA; qPCR, quantitative PCR.

H2A.Z has been known to play an important role in keeping genome stability of eukaryotic cells and has two isoforms that are different only in three amino acids of the protein. To further study isoform-specific functions of H2A.Z, we tried to find unique siRNAs depleting each isoform of H2A.Z exclusively and compared our depletion effect with those of previous reported siRNAs (Fig. S1, *A* and *B*). Our siRNAs, H2AFV-2 and H2AFZ-1, could deplete H2A.Z transcripts efficiently but selectivity was not good. On the contrary, the previous reported siRNAs, H2AFV-4 and H2AFZ-2, could deplete each isoform of H2A.Z efficiently and exclusively. Thus, we decided to carry out our studies with H2AFV-4 and H2AFZ-2 oligos (Fig. 1). Protein abundance was also reduced consistently by siRNA depletion but it was not clear how much fraction of the remaining H2A.Z protein after RNAi resulted from H2AFV or H2AFZ since the antibody specificity to two isoforms is not clear (Fig. S1*C*).

**Figure 2:**
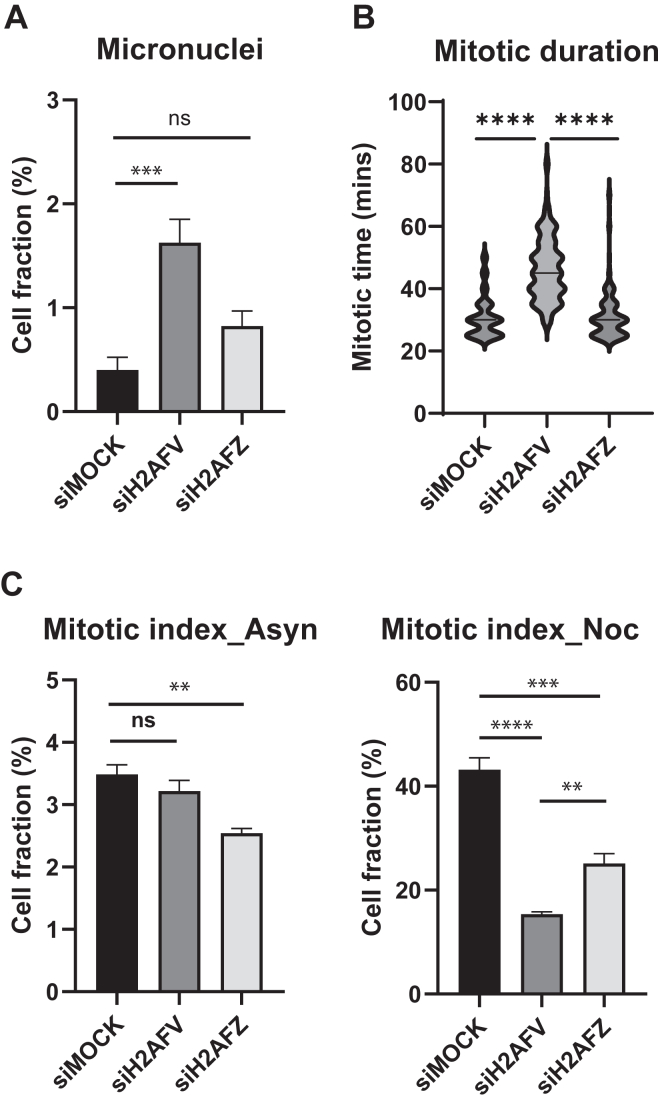
**Mitotic defects by H2A.Z RNAi**. *A*, chromosome segregation integrity of RNAi cells. HeLa Tet-on cells were transfected by siRNA oligos targeting H2AFV or H2AFZ for 48 h in 96-well imaging plates. Cells were fixed and DNAs were stained by Hoechst 33342. Cells with micronuclei were counted and mean values (n = 3) with standard deviations were shown. Two-tailed *p* value was calculated by unpaired student *t* test. ∗∗∗∗ means *p* value is less than 0.0001, ∗∗∗ less than 0.001, and ∗∗ less than 0.01. NS stands for not significant. *B*, mitotic duration of RNAi cells. HeLa Tet-on cells expressing H2B-mCh were first transfected by siRNA oligos targeting H2AFV or H2AFZ for 24 h in 96-well imaging plates and then arrested by thymidine for 20 h. Cells were released into mitosis with live-cell imaging. Mitotic time was counted from NEBD (nuclear envelope breakdown) to anaphase (sister chromatid separation). Cells from three wells (n = 150) were combined and their mitotic time was shown by violin plot. *C*, mitotic index of RNAi cells. HeLa Tet-on cells were transfected by siRNA oligos targeting H2AFV or H2AFZ for 32 h. Cells were then incubated with DMSO (*left*) or nocodazole (*right*) for 16 h. Cells were fixed and stained by phospho-specific antibody against histone H3pS10 and Hoechst 33342. 4N DNA and H3pS10 positive cells were counted as mitotic cells. Mean values with standard deviations (n = 3) were shown. DMSO, dimethyl sulfoxide.

We next studied if depletion of H2A.Z isoforms affects cell cycle progression. H2A.Z was known to regulate cyclin expression, and H2AFZ depletion arrested cells specifically at G1 phase (11, 28). After 2 days of siRNA depletion, we examined DNA content by fluorescence-activated cell sorting (FACS) and analyzed cell cycle profile by univariate modeling of FlowJo software (Fig. S2*A*). We observed slight increase of G1 fraction after H2AFZ depletion but overall cell cycle profiles were quite similar to each other. We then examined if chromosome segregation fidelity was reduced after H2A.Z isoform depletion by counting cells with micronuclei since micronuclei mostly resulted from chromosome missegregation (Figs. 2*A* and S3*A*). Consistent with the earlier report (11), H2AFV depletion increased micronuclei fraction but H2AFZ depletion did not, indicating a specific role of H2AFV in chromosome segregation. To verify chromosome segregation fidelity during mitosis, we carried out live cell imaging using H2B-mCherry stable lines after H2A.Z isoform-specific RNAi. H2AFV RNAi cells stayed in mitosis for a significantly longer time but H2AFZ RNAi cells did not (Fig. 2*B*). Cells staying longer in mitosis after H2AFV RNAi exhibited mostly inefficient chromosome alignment in metaphase, suggesting a specific defect in chromosome segregation (Fig. S4).

### DNA damages and centromeric transcription defect by H2AFV depletion

**Figure 3:**
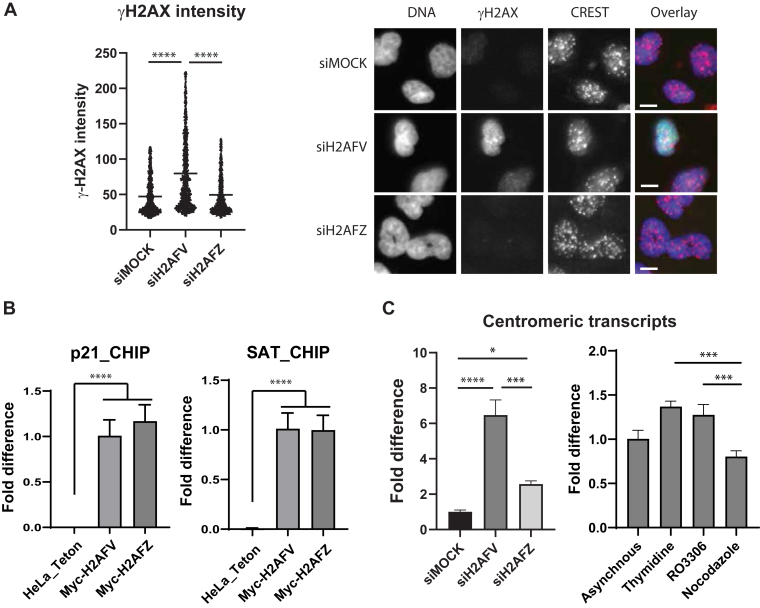
**DNA damage repair and centromeric transcription defects by H2A.Z RNAi.***A*, DNA damages of RNAi cells. HeLa Tet-on cells were transfected by siRNA oligos targeting H2AFV or H2AFZ for 48 h in 96-well imaging plates. Cells were fixed and stained by phospho-specific antibody against histone H2A.XpS139 (γH2AX) and Hoechst 33342. Cells from three wells (n = 1000) were combined and their γH2AX levels were shown by violin plot (*left*). Representative images were shown (*right*). The scale bar represents 10 μm. Two-tailed *p* value was calculated by unpaired student *t* test. ∗∗∗∗ means *p* value is less than 0.0001, ∗∗∗ less than 0.001, and ∗∗ less than 0.01. NS stands for not significant. *B*, ChIP of Myc-H2AFV and Myc-H2AFZ. HeLa Tet-on cells, Myc-H2AFV cells, or Myc-H2AFZ cells were processed for immunoprecipitation by Myc antibody. Immunoprecipitated fraction was further processed for qPCR with primers annealing p21 promoter sequence to examine noncentromeric DNA level (*left*) or primers annealing chromosome 13/21 alpha satellite sequence to examine centromeric DNA level (*right*). Mean values with standard deviations (three technical replicates) were shown. Two biological replicates showed similar results. *C*, centromeric RNA levels of RNAi cells. HeLa Tet-on cells were transfected by siRNA oligos targeting H2AFV or H2AFZ for 48 h (*left*), or cells were incubated with DMSO (asynchronous), thymidine (G1 arrest), RO3306 (G2 arrest), or nocodazole (M arrest) for 16 h (*right*). cDNAs were synthesized from total RNAs of the cells and used for qPCR with primers annealing chromosome 13/21 alpha satellite sequence to examine centromeric RNA levels. Mean value of RNA levels (three technical replicates) was shown with standard deviation. Two biological replicates showed a similar trend. cDNA, complementary DNA; ChIP, chromatin immunoprecipitation; DMSO, dimethyl sulfoxide; qPCR, quantitative PCR.

Next, we tested if the SAC integrity was compromised by H2A.Z isoform-specific depletion (Figs. 2*C* and S3*B*). In asynchronous culture, the mitotic fraction of H2AFV depleted cells was similar to that of control cells, consistent with cell cycle profile data by FACS analysis. However, when cells were challenged with nocodazole overnight, the mitotic fraction significantly diminished by H2AFV or H2AFZ depletion, suggesting SAC maintenance defect. Furthermore, subG2-DNA content of cells accumulated noticeably (Fig. S2*B*). H2A.Z has been previously implicated in DNA damage repair (15, 16, 17). We then wondered if H2A.Z depletion resulted in increased DNA damages which might interfere cell cycle progression. Thus, we examined gamma-H2AX intensity, a marker of double-stranded DNA break, in H2A.Z depleted cells (Figs. 3*A* and S7*B*). Interestingly, H2AFV depletion increased gamma-H2AX level significantly but H2AFZ depletion did not. Furthermore, we observed that some of gamma-H2AX signal overlapped with the centromere marker, CREST, suggesting specific DNA damages at the centromere (Figs. 3*A* and S5). In this regard, DNA damages often occur at the centromere during replication or transcription due to its highly repeated sequences and secondary structures (29, 30).

H2A.Z localizes to centromeric chromatin in mammalian cells and its yeast ortholog regulates centromeric silencing (12, 25). Therefore, we wondered whether both isoforms localize to the centromeric chromatin of human cells and regulates centromeric transcription. For this, we performed chromatin immunoprecipitation (ChIP) assays using a cell line expressing Myc-H2AFV or Myc-H2AFZ under a doxycycline-inducible promoter (Figs. 3*B* and S7*C*). First, we examined the noncentromeric DNA localization of Myc-H2AFV and Myc-H2AFZ by ChIP with p21 promoter-specific primers (10, 31). Both isoforms were clearly localized at the noncentromeric DNA region. Next, we investigated their centromeric DNA localization. The level of centromeric DNA from chromosome 13/21, detected using primers targeting the alpha satellite sequences of chromosome 13 and 21, was enriched in the Myc-H2AFV or Myc-H2AFZ immunoprecipitated fractions. This indicates that both isoforms of H2A.Z localized to the centromere. When we examine centromeric transcription, we found that depletion of H2AFV isoform strongly increased centromeric transcripts, suggesting an inhibitory role of H2AFV on centromeric transcription. Additionally, we analyzed centromeric transcription across different cell cycle stages. Centromeric transcription was slightly upregulated when cells were arrested in G1 by thymidine, but the differences across cell cycle stages appeared to be minor (Figs. 3*C* and S6*A*).

### H2A.Z isoforms interact with mitotic kinases for its functional regulation

**Figure 4:**
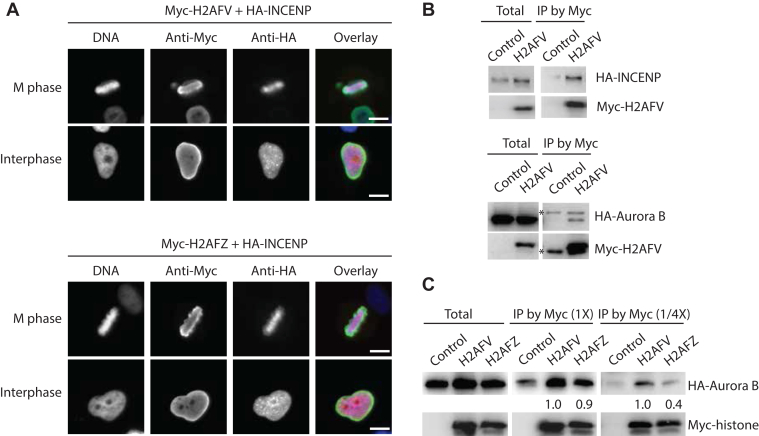
**Subcellular localization and protein interaction of H2A.Z and INCENP**. *A*, immunofluorescence of Myc-H2A.Z and HA-INCENP expressing cells. HeLa Tet-on cells were first transfected by Myc-H2AFV or Myc-H2AFZ together with HA-INCENP plasmids for 24 h in 96-well imaging plates. Cells were fixed and stained by Myc or HA antibody and Hoechst 33342. Representative cell images were shown. The scale bar represents 10 μm. *B*, CoIP experiment of INCENP or Aurora B with H2A.Z. HA-INCENP or HA-Aurora B was cotransfected with empty vector or Myc-H2AFV into HeLa Tet-on cells and collected at M-phase by nocodazole treatment for 16 h. Myc-tagged histones were pulled down by Myc beads. Total lysates and immunoprecipitated fractions were resolved by SDS-PAGE and processed for Western blot. ∗ indicates immunoglobulin heavy chain in HA-Aurora B blot and immunoglobulin light chain in Myc-H2AFV blot. *C*, CoIP experiment of Aurora B with histones. HA-Aurora B was cotransfected with empty vector, Myc-H2AFV, or Myc-H2AFZ into HeLa Tet-on cells and arrested at M-phase with nocodazole for 16 h. Myc-histones were pulled down by Myc beads. Total lysates and immunoprecipitated fractions were resolved by SDS-PAGE and processed for Western blot. Normalization of coimmunoprecipitated signals by relevant Myc-histone signals was carried out by ImageJ gel quantitation method and the result was shown. CoIP, co-immunoprecipitation; HA, hemagglutinin.

Previously, H2A.Z was shown to localize across the entire chromatin, including pericentromeric chromatin, and bind to the pericentromeric heterochromatin-binding protein, inner centromere protein (INCENP) (5, 12, 13). Furthermore, H2AFV depletion reduces the level of CPC, which consists of INCENP and Aurora B, on mitotic chromosomes (11). We therefore examined the subcellular localization of the two isoforms of H2A.Z and INCENP by transient transfection (Fig. 4*A*). Both H2A.Z isoforms localized to the nucleoplasm during interphase and the entire chromosome during M phase, with no observable differences in their localization patterns. INCENP localized to the nucleoplasm and heterochromatin during interphase, consistent with earlier studies (32, 33). During mitosis, it was enriched at the middle region of metaphase chromosomes, most likely corresponding to the inner centromeres. Thus, both H2A.Z isoforms and INCENP localized to similar regions of chromatin. Aurora B, a subunit of CPC, also exhibited a localization pattern similar to that of INCENP (Fig. S8). To verify that the diffuse localization of H2A.Z across the entire chromosome was not an artifact of transient transfection and overexpression, we constructed a cell line expressing H2AFV-SNAP under the endogenous promoter by integrating the SNAP gene into the genomic locus of H2AFV gene. Using the click-it method, we observed a similar subcellular localization of H2AFV-SNAP (data not shown), confirming that the diffuse chromosomal localization is not an artifact of transient transfection.

Next, we investigated whether INCENP or Aurora B binds to the H2AFV isoform to regulate its function. To address this, we performed coimmunoprecipitation experiments. After transiently expressing Myc-H2AFV with hemagglutinin (HA)-Aurora B or Myc-H2AFV with HA-INCENP in HeLa cells, we immunoprecipitated Myc-H2AFV using a Myc antibody and examined whether HA-Aurora B or HA-INCENP was coimmunoprecipitated with Myc-H2AFV. Both INCENP and Aurora B was efficiently coimmunoprecipitated with H2AFV (Fig. 4*B*). We then tested whether Aurora B exhibits differential binding to the two H2A.Z isoforms. Similar coimmunoprecipitation experiments with H2A, H2AFV, or H2AFZ, showed that both H2A.Z isoforms bind Aurora B with comparable affinity when overexpressed (Fig. S7*A*). To further explore potential differences in binding affinity, we reasoned that reducing the total protein concentration of the cell lysates might weaken low-affinity protein interactions. Therefore, after preparing a total cell lysate, we divided it into two fractions, one at regular concentration and the other diluted to one-fourth of the original concentration. Using this approach, we observed stronger binding between H2AFV and Aurora B compared to that between H2AFZ and Aurora B. This suggests that Aurora B likely targets H2AFV specifically to regulate its mitotic functions (Fig. 4*C*).

**Figure 5:**
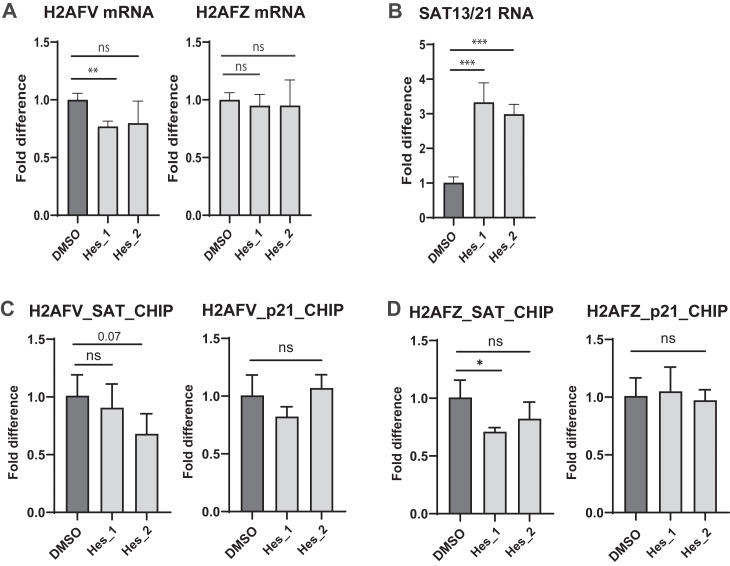
**Regulation of H2A.Z by mitotic kinases**. *A*, mRNA levels of drug-treated cells. HeLa Tet-on cells were treated with hesperadin for 24 h. cDNAs were synthesized from total RNAs of the cells and used for qPCR to examine H2AFV mRNA level (*left*) and H2AFZ mRNA level (*right*). Mean values of mRNA levels (three technical replicates) were shown with standard deviations. Two biological replicates of drug-treated samples were also shown. Two-tailed *p* value was calculated by unpaired student *t* test. ∗∗∗∗ means *p* value is less than 0.0001, ∗∗∗ less than 0.001, and ∗∗ less than 0.01. ns stands for not significant. *B*, centromeric RNA levels of drug-treated cells. HeLa Tet-on cells were treated with hesperadin for 24 h. cDNAs were synthesized from total RNAs of the cells and used for qPCR with primers annealing chromosome 13/21 alpha satellite sequence to examine centromeric RNA levels. Mean values of mRNA levels (three technical replicates) was shown with standard deviations. Two biological replicates of drug-treated samples were also shown. *C* and *D*, ChIP of Myc-H2AFV (*C*) or Myc-H2AFZ (*D*) in drug-treated cells. HeLa Tet-on cells expressing Myc-H2AFV were treated with hesperadin for 24 h. Immunoprecipitated fraction by Myc antibody was processed for qPCR with primers annealing chromosome 13/21 alpha satellite sequence or p21 promoter sequence. Mean values of centromeric DNA levels (three technical replicates) were shown with standard deviations. Two biological replicates of drug-treated samples were also shown. cDNA, complementary DNA; ChIP, chromatin immunoprecipitation; qPCR, quantitative PCR.

Next, we investigated whether inhibition of Aurora B by small-molecule inhibitors would lead to functional defects of H2AFV. We first examined the abundance of H2A.Z isoforms after treatment with Aurora B kinase inhibitors (Figs. 5*A* and S6*B*). The H2AFV mRNA level was slightly decreased by the Aurora B inhibitor, hesperidin, whereas the H2AFZ mRNA level remained unchanged. However, the H2A.Z protein abundance, as assessed by an H2A.Z antibody, was not altered by treatment with Aurora B inhibitors (Fig. S6*B*). We then explored whether centromeric transcription was influenced by Aurora B inhibition (Fig. 5*B*). Interestingly, centromeric transcription was significantly increased following Aurora B inhibition. To determine whether the centromeric localization of Myc-H2AFV or Myc-H2AFZ was affected by kinase inhibition, we performed ChIP experiment (Fig. 5, *C* and *D*). Aurora B inhibition slightly reduced the centromeric level of Myc-H2AFZ in one of two biological replicates, but no significant difference in localization was observed overall. These ChIP results suggest that abnormal centromeric transcription following kinase inhibition is likely due to altered downstream signaling of H2A.Z at the centromere, rather than changes in its centromeric localization. Collectively, our data support the idea that H2A.Z isoforms play distinct roles in ensuring accurate chromosome segregation by maintaining proper DNA damage repair and regulating centromeric transcription.

## Discussion

We have investigated the isoform-specific role of H2A.Z in chromosome segregation and identified that the H2AFV isoform specifically protects centromeres against double-stranded DNA breaks and regulates centromeric transcription. Furthermore, we demonstrated that the kinase, Aurora B, interact with H2AFV isoform and regulate centromeric transcription, potentially through its role in inducing epigenetic changes of the H2AFV isoform.

Two isoforms of H2A.Z exhibit both redundant and nonredundant functions. For instance, H2AFV cannot compensate for the embryonic development defects observed in H2AFZ KO mice, whereas the intestinal deletion of either isoform does not result in noticeable defects (34, 35). Structural study revealed that the L1 loop of the two isoforms undergo significant conformation changes due to an amino acid difference at position 38 (36). This structural difference may contribute to variations in binding affinity to certain partners, leading to their nonredundant functions.

We also observed distinct differences in some mitotic functions of the isoforms, which may be regulated by their interactions with the mitotic kinase Aurora B. Aurora B plays an essential role in mitosis, including establishment of chromosome biorientation. When microtubules fail to attach properly to sister chromatids, Aurora B facilitates the formation of mitotic checkpoint complex, which inhibits transition from metaphase to anaphase (37, 38). Additionally, Aurora B has been shown to phosphorylate histone H3 at serine 10 (H3S10) to promote chromatin condensation and at serine 28 (H3S28) to regulate gene expression (39, 40). Thus, the increase in centromeric transcription following Aurora B inactivation may result either indirectly from deregulation of other factors or directly from alterations in H2AFV-related epigenetic modifications (Fig. S9). Further studies on how Aurora B regulates centromeric transcription would be highly valuable. In this context, H2A.Z has previously been shown to be phosphorylated at multiple sites (7). It would be interesting to investigate whether Aurora B phosphorylates the H2AFV isoform to regulate its function, similar to its role in histone H3 phosphorylation.

Highly repetitive nature of centromeric sequences and their secondary structures such as R-loop often complicates centromeric replication by causing issues like replication fork stalling (30). Therefore, the timely resolution of these complications is critical for genome stability, as its dysregulation can lead to double-stranded DNA breaks and chromosome arm translocations, which are hallmarks of tumors (29). In this study, we demonstrated that H2AFV deletion specifically increases double-stranded DNA breaks at the centromere and enhances centromeric transcription. We propose that increased centromeric transcription may interfere with centromere stability, resulting in double-stranded DNA breaks (Fig. S9). Since H2A.Z has been shown to regulate the formation of highly folded secondary chromatin structures in conjunction with HP1 alpha (26), structures essential for various cellular processes like replication, transcription, or DNA damage repair, the depletion of H2AFV naturally destabilizes these chromatin structures and further disrupts chromatin dynamics. In this regard, H2A.Z is known to be recruited to transcription start sites to establish nucleosome-free regions for transcription regulation (7). Therefore, H2AFV inhibition may dysregulate centromeric transcription, leading to replication fork stalling and subsequent DNA damage. Conversely, an opposite scenario such as increased DNA damages may indirectly enhance centromeric transcription. As H2AFV has been shown to localize to DNA damage sites to facilitate repair (16), its depletion would result in inefficient DNA repair at the centromere, potentially increasing centromeric transcription as suggested by a recent report (41). Therefore, it is of great interest to further investigate how H2AFV protects centromeric chromatin integrity in future studies.

## Experimental procedures

### Cell culture and transfection

HeLa Tet-on cells (Clontech) were grown at 37 °C in Dulbecco's modified Eagle's medium (Gibco) supplemented with 10% fetal bovine serum (Sorfa) in a humid atmosphere with 5% CO_2_. For cell cycle arrest, cells were treated with 2 mM thymidine (Sigma-Aldrich), 10 μM RO3306 (APExBIO), 330 nM nocodazole (Sigma-Aldrich) for 14 to 18 h. For kinase inhibition, cells were treated with 3 μM AZ3046 (APExBIO) or 100 nM hesperadin (Sigma-Aldrich) for 24 h. For the generation of stable cell lines, HeLa Tet-On cells were transfected with pTRE2-hyg vectors encoding siRNA-resistant WT Myc-H2AFV or Myc-H2AFZ transgenes. Clones of cells were selected in regular media containing 300 μg/ml hygromycin (MesGen) and maintained in media with 100 μg/ml hygromycin. Subsequently, 5 μg/ml doxycycline (MesGen) was used for induction of Myc-H2A.Z isoform expression. For RNAi experiment, cells were transfected with siRNA oligonucleotides (GenePharma) using Lipofectamine RNAiMAX (Invitrogen) for 24 to 48 h. The sequences of the H2AFV-4 siRNAs were 5′- GUGACAGUUGUGUGUUGAU -3′. The sequences of the H2AFZ-2 siRNAs were 5′- GCCGUAUUCAUCGACACCU -3′. Plasmid transfection was carried out by Effectene reagent (Qiagen) according to the manufacturer’s instructions.

### Antibodies and immunoprecipitation

The following primary antibodies were used for immunofluorescence: anti-γH2AX (CWBIO, 1:500), CREST (, 1:100), anti-Myc (Proteintech, 1:1000), goat-anti-rabbit IgG FITC conjugated (CWBIO, 1:300), goat-anti-human IgG FITC conjugated (CWBIO, 1:300), and goat-anti-mouse IgG TRITC conjugated (CWBIO, 1:300). The following primary antibodies were used for Western blot: anti-Myc (Proteintech, 1:3000), anti-HA (Roche, 1:1000), anti-GAPDH (MesGen, 1:3000), anti-H3pS10 (Cell Signaling, 1:1000), anti-H2AZ (Proteintech, 1:1000), goat anti-mouse IgG HRP conjugate (Proteintech, 1:3000), and goat anti-rabbit IgG HRP conjugate (Proteintech, 1:3000).

Mitotic cells coexpressing HA tagged gene and Myc tagged gene were resuspended in lysis buffer (25 mM Tris–HCl, pH 7.4, 150 mM KCl, 1% NP-40, 1 mM EDTA, and 5% glycerol) supplemented with protease inhibitor cocktail (MesGen) and phosphatase inhibitor cocktail (MesGen) and sonicated for 20 s with intervals on ice. The supernatants were incubated with Anti-c-Myc magnetic beads (Pierce) for 2 h at 4 °C with rotation. The beads were washed with lysis buffer, and proteins bound to beads were released with SDS loading buffer, resolved in SDS-PAGE, and analyzed by Western blotting.

### Immunofluorescence, imaging, and FACS

Cells grown on 96-well imaging plate (Corning) were fixed by 4% paraformaldehyde in PHEM buffer at room temperature for 10 min. Cells were then permeabilized with PBST (phosphate buffered saline containing 0.1% Triton X-100) and blocked with PBST containing 3% bovine serum albumin for 30 min. Primary antibodies were incubated at room temperature for 2 h and secondary antibodies for 1 h. DNA was stained by Hoechst 33342 (1 μg/ml) for 5 min. Cell images were acquired by a 20× objective lens in a Nikon Eclipse Ti-E microscope and processed with ImageJ (NIH). For γH2AX intensity measurement, cells on images were segmented with DNA intensity using ImageJ software, and then γH2AX intensity from each nucleus was extracted computationally.

For time-lapse imaging, HeLa Tet-on H2B-mCherry cells were first arrested at G2 by 10 μΜ RO3306 for 16 h and released with recording every 10 min for a total duration of 24 h with a 10× objective in a Nikon Eclipse Ti-E microscope equipped with a temperature- and CO_2_-controlled stage incubation unit (Okolab).

For FACS analysis, cells grown on 6-well plate were resuspended in cold 70% ethanol and fixed at −20 °C overnight. Cells were then washed with PBS and incubated with primary antibody for 2 hours. Cells were washed with PBS and incubated with secondary antibody for 30 min. Cells were then washed with PBS, stained with Hoechst 33342 (1 μg/ml) for 5 min, and analyzed with FACS.

### Reverse transcription - quantitative PCR and ChIP

Total RNA was isolated from cells using RNAiso Plus (Takara Bio) according to the manufacturer's instructions. RNA was transcribed using PrimeScript reverse transcriptase (Takara Bio) according to the manufacturer's instructions. Quantitative real-time PCR was carried out using TB Green PreMix Ex Taq (Takara Bio) according to the manufacturer's instruction in CFX96 Real Time PCR machine (Bio-Rad). Primer sequences for quantitative PCR (qPCR): GAPDH_F: 5′-TGA TGA CAT CAA GAA GGT GGT GAA G-3′, GAPDH-R: 5′-TCC TTG GAG GCC ATG TGG GCC AT-3′, H2AFV_F: 5′V_R: 5′-GCGAGTCTTCAAGTGTCTGT-3′, H2AFZ_F: 5′ CTGCCTTGCTTGCTTGAGC-3′, H2AFZ_R: 5′ GGATGGCTGCGCTGTAC-3′, SAT13/21_F: 5′-TAG ACA GAA GCA TTC TCA GAA ACT-3′, SAT13/21_R: 5′-TCC CGC TTC CAA CGA AAT CCT CCA AAC-3′, p21_F: 5′-CTGTGGCTCTGATTGGCTTT-3′, p21_R: 5′-CTCCTACCATCCCCTTCCTC-3.

For ChIP, cells were cross-linked with 1% formaldehyde (Sinopharm) in cell culture media for 15 min at room temperature. The reaction was stopped by adding 125 mM glycine (MesGen) in cell culture media. Scraped cells were washed by cold PBS two times. Cells were lysed for 10 min on ice in lysis buffer A [5 mM Pipes pH 8.0, 85 mM KCl, 0.5% NP40, 0.5 mM PMSF, and protease inhibitor cocktail (MesGen)]. Nuclei were then collected by centrifugation at 2000 rpm for 10 min at 4 °C and resuspended in lysis buffer B [50 mM Hepes pH 7.9, 140 mM NaCl, 1 mM EDTA, 1% Triton X-100, 0.1% sodium deoxycholate, 0.1% SDS, 0.5 mM PMSF, and protease inhibitor cocktail (MesGen)]. Chromatin was fragmented to an approximate length of 500 bps using a Scientz sonifier. Supernatant were collected after centrifugation at 12,000*g* for 10 min at 4 °C. Anti-c-Myc magnetic beads (Pierce) were washed with 5 mg/ml bovine serum albumin in PBS two times and incubated with supernatant for 6 h at 4 °C. Beads were washed two times each with wash buffer A (20 mM Tris–HCl pH8, 0.15 M NaCl, 1 mM EDTA, 0.1% SDS, and 1% Triton X-100), wash buffer B (20 mM Tris–HCl pH 8, 0.5 M NaCl, 1 mM EDTA, 0.1% SDS, and 1% Triton X-100), wash buffer C (20 mM Tris–HCl pH 8, 0.25 M LiCl, 1 mM EDTA, 1% NP40, and 0.5% sodium deoxycholate) and TE buffer (10 mM Tris–HCl pH 8, and 1 mM EDTA). Chromatin was eluted in elution buffer (10 mM Tris–HCl pH8, 0.3 M NaCl, 5 mM EDTA, 0.5% SDS, and 10 μg/ml RNase A) and cross-link was reversed at 65 °C overnight. Proteins were digested by adding 200 μg/ml proteinase K at 55 °C for 4 h. DNA was purified using the QIAquick PCR Purification Kit (Qiagen) and eluted in 50 μl TE buffer. ChIP samples were analyzed by qPCR.

## Data availability

All data are contained within the manuscript. Any inquiries regarding the data should be directed to the corresponding author, Jungseog Kang, jsk16@nyu.edu.

## Supporting information

This article contains supporting information.

## Conflict of interest

The authors declare that they have no conflicts of interest with the contents of this article.

## Supporting information

Sup Figure 1

Sup Figure 2

Sup Figure 3

Sup Figure 4

Sup Figure 5

Sup Figure 6

Sup Figure 7

Sup Figure 8

Sup Figure 9
