# Supplementary material for: Role of noncanonical histone H2A variant, H2A.Z, to maintain proper centromeric transcription and chromosome segregation
Source: J Biol Chem. 2025 Mar 28;301(5):108464. doi: 10.1016/j.jbc.2025.108464 (PMC12051535; doi:10.1016/j.jbc.2025.108464)
Supplement: Sup Figure 1 [file mmc1.pdf]

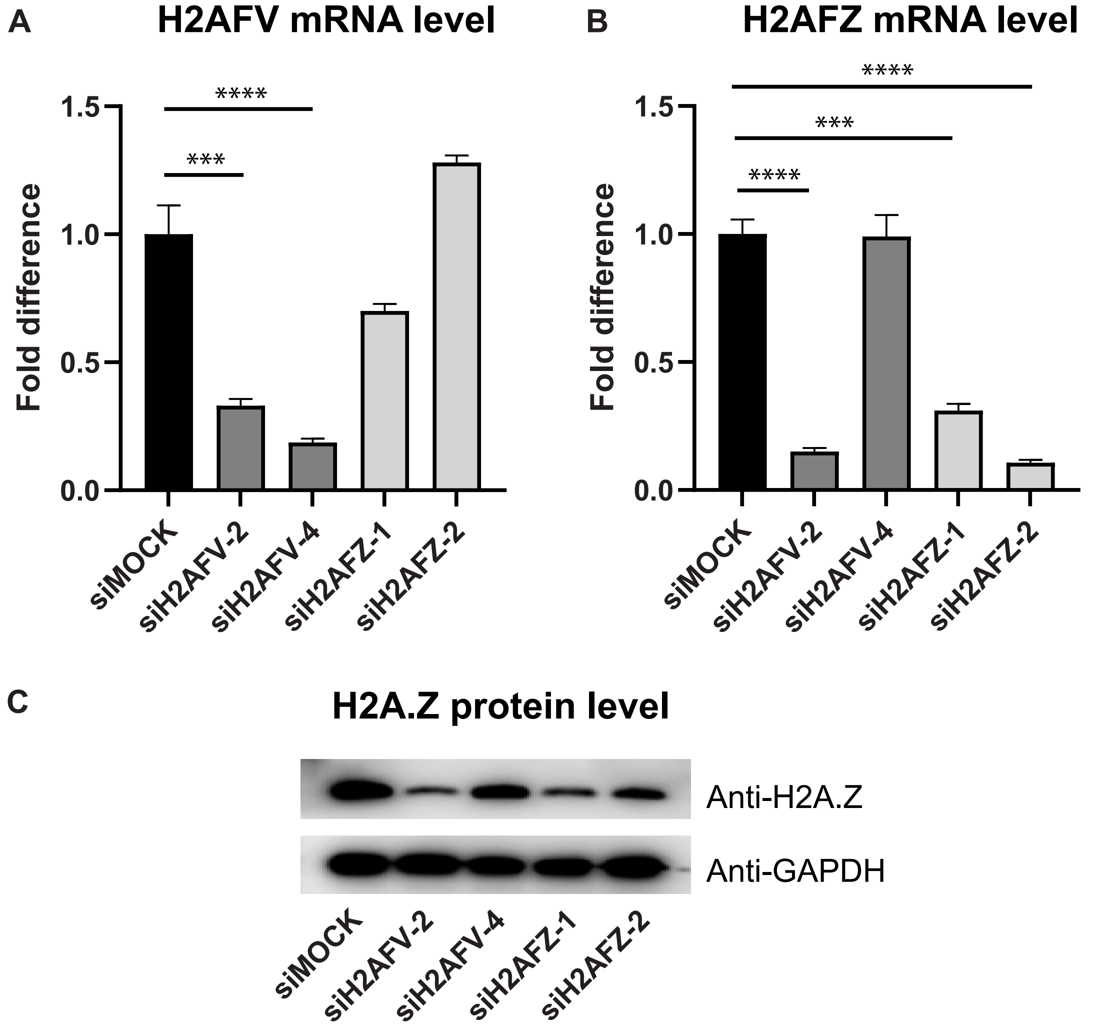

Sup Figure 1. Isoform-specific depletion by different H2A.Z RNAi oligos. (A and B) mRNA levels of RNAi cells. HeLa Tet-on cells were transfected by several different siRNA oligos targeting H2AFV or H2AFZ for 48 hrs. cDNAs were synthesized from total RNAs of the cells and used for qPCR to examine their mRNA levels. Mean values of mRNA levels (three technical replicates) were shown with standard deviations. Two biological replicates showed a similar trend. Two-tailed P value was calculated by unpaired student t-test. **** means P value is less than 0.0001, *** less than 0.001, and ** less than 0.01. NS stands for not significant. (C) Protein abundance of RNAi cells. HeLa Tet-on cells were transfected by several different siRNA oligos targeting H2AFV or H2AFZ for 48 hrs. Total lysates were resolved by SDS-PAGE and processed for western blot.
