# Supplementary material for: Role of noncanonical histone H2A variant, H2A.Z, to maintain proper centromeric transcription and chromosome segregation
Source: J Biol Chem. 2025 Mar 28;301(5):108464. doi: 10.1016/j.jbc.2025.108464 (PMC12051535; doi:10.1016/j.jbc.2025.108464)
Supplement: Sup Figure 2 [file mmc2.pdf]

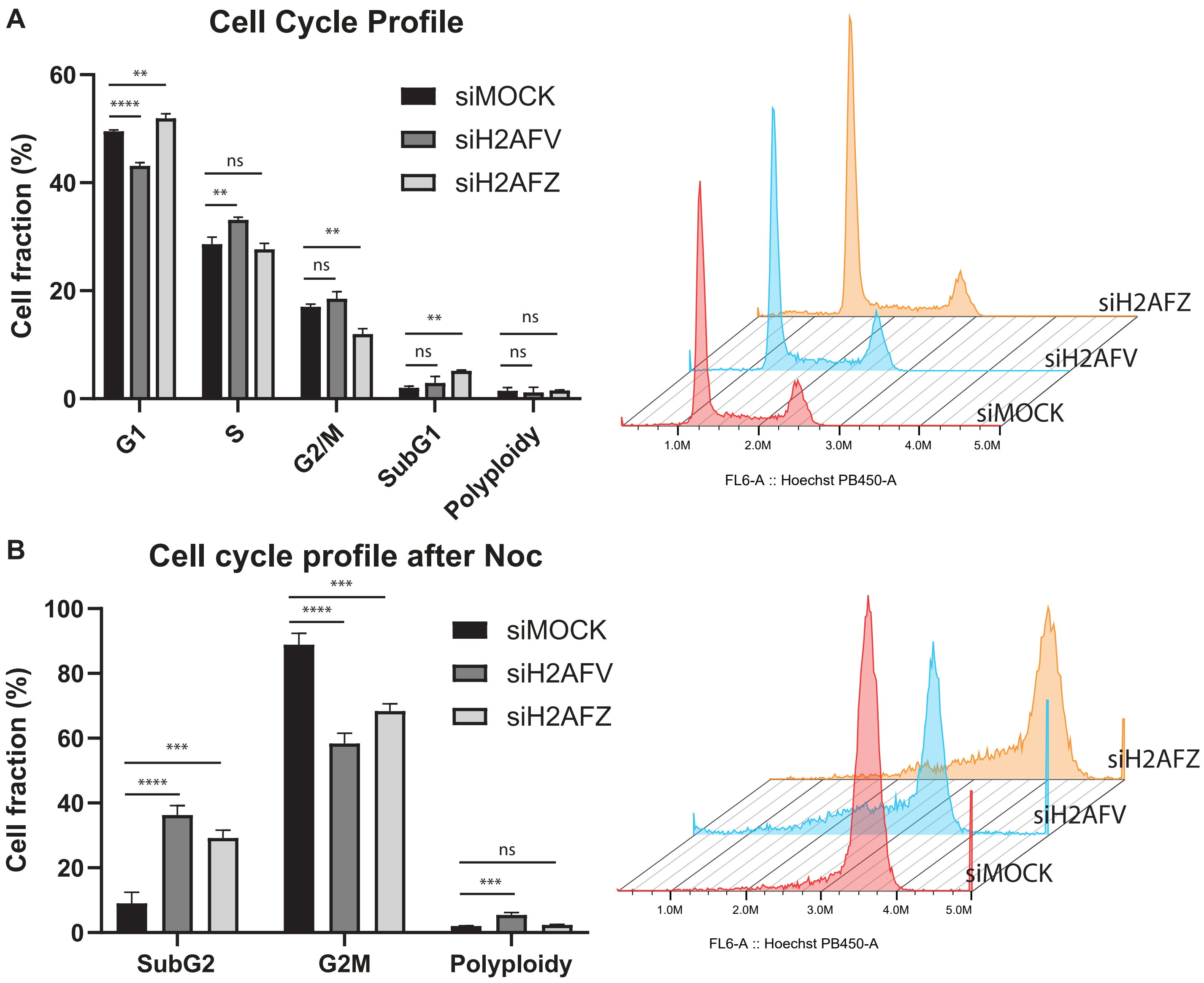

Sup Figure 2. Cell cycle profile of H2A.Z RNAi cells. (A and B) FACS analysis of RNAi cells. HeLa Tet-on cells were transfected by siRNA oligos targeting H2AFV or H2AFZ for 32 hrs. Cells were then incubated with DMSO (A) or nocodazole (B) for 16 hrs. Cells were fixed and stained by Hoechst 33342. Cell cycle is analyzed by univariate modeling of FlowJo software. Mean value with standard deviation (n = 3) were shown. Two-tailed P value was calculated by unpaired student t-test. **** means P value is less than 0.0001, *** less than 0.001, and ** less than 0.01. NS stands for not significant.
