# Supplementary material for: Role of noncanonical histone H2A variant, H2A.Z, to maintain proper centromeric transcription and chromosome segregation
Source: J Biol Chem. 2025 Mar 28;301(5):108464. doi: 10.1016/j.jbc.2025.108464 (PMC12051535; doi:10.1016/j.jbc.2025.108464)
Supplement: Sup Figure 3 [file mmc3.pdf]

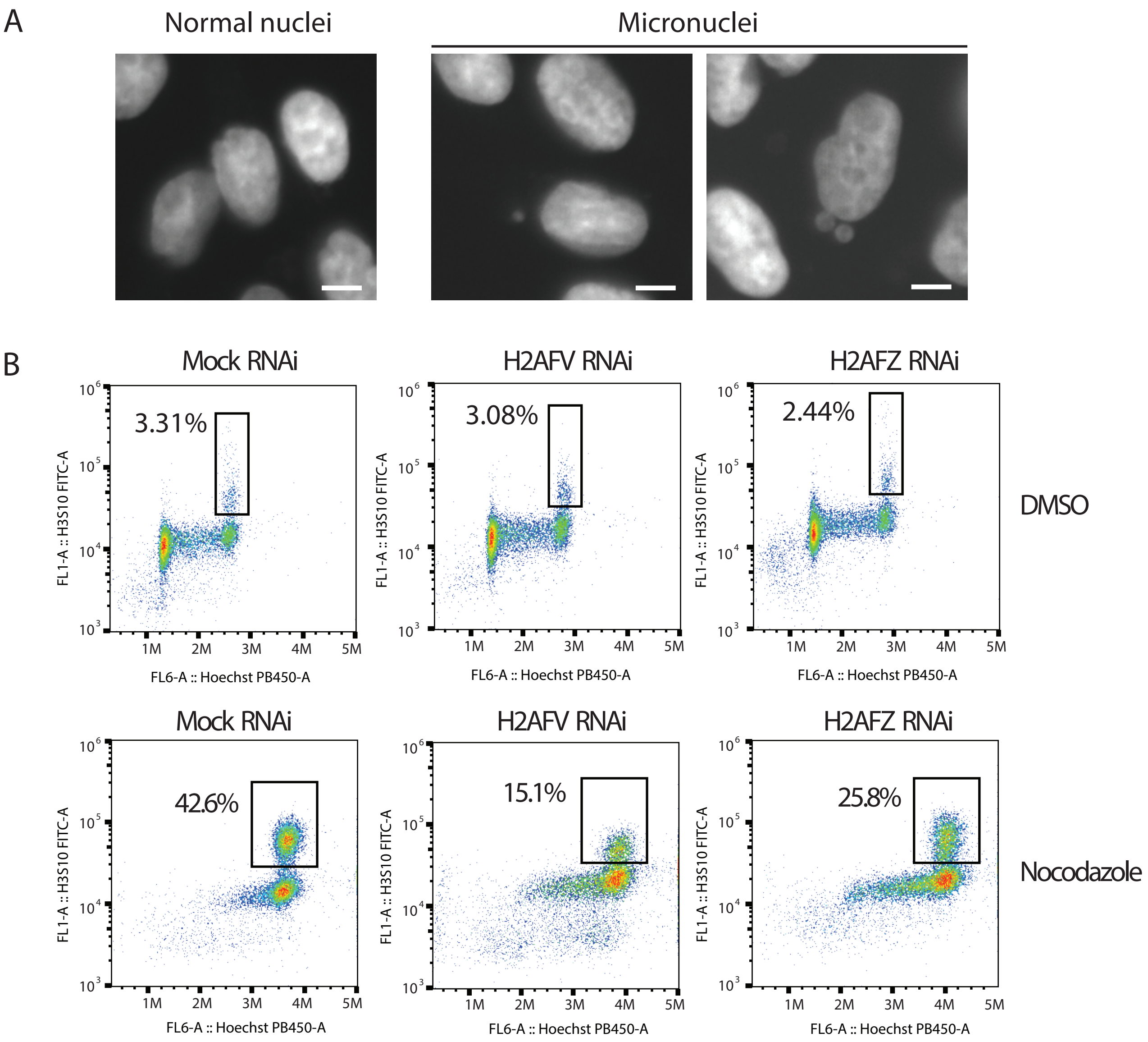

Sup Figure 3. Chromosome missegregation and mitotic index after H2A.Z RNAi. (A) Representive images of micronuclei. HeLa Tet-on cells were transfected by siRNA oligos targeting H2AFV or H2AFZ for 48 hrs in 96-well imaging plates. Cells were fixed and DNAs were stained by Hoechst 33342. Bar, 10 µm. (B) Mitotic index of RNAi cells. HeLa Tet-on cells were transfected by siRNA oligos targeting H2AFV or H2AFZ for 32 hrs. Cells were then incubated with DMSO or nocodazole for 16 hrs. Cells were fixed and stained by phospho-specific antibody against H3pS10 and Hoechst 33342. Representative FACS results showing 4N DNA and H3pS10 positive mitotic cells were shown.
