# Supplementary material for: Role of noncanonical histone H2A variant, H2A.Z, to maintain proper centromeric transcription and chromosome segregation
Source: J Biol Chem. 2025 Mar 28;301(5):108464. doi: 10.1016/j.jbc.2025.108464 (PMC12051535; doi:10.1016/j.jbc.2025.108464)
Supplement: Sup Figure 4 [file mmc4.pdf]

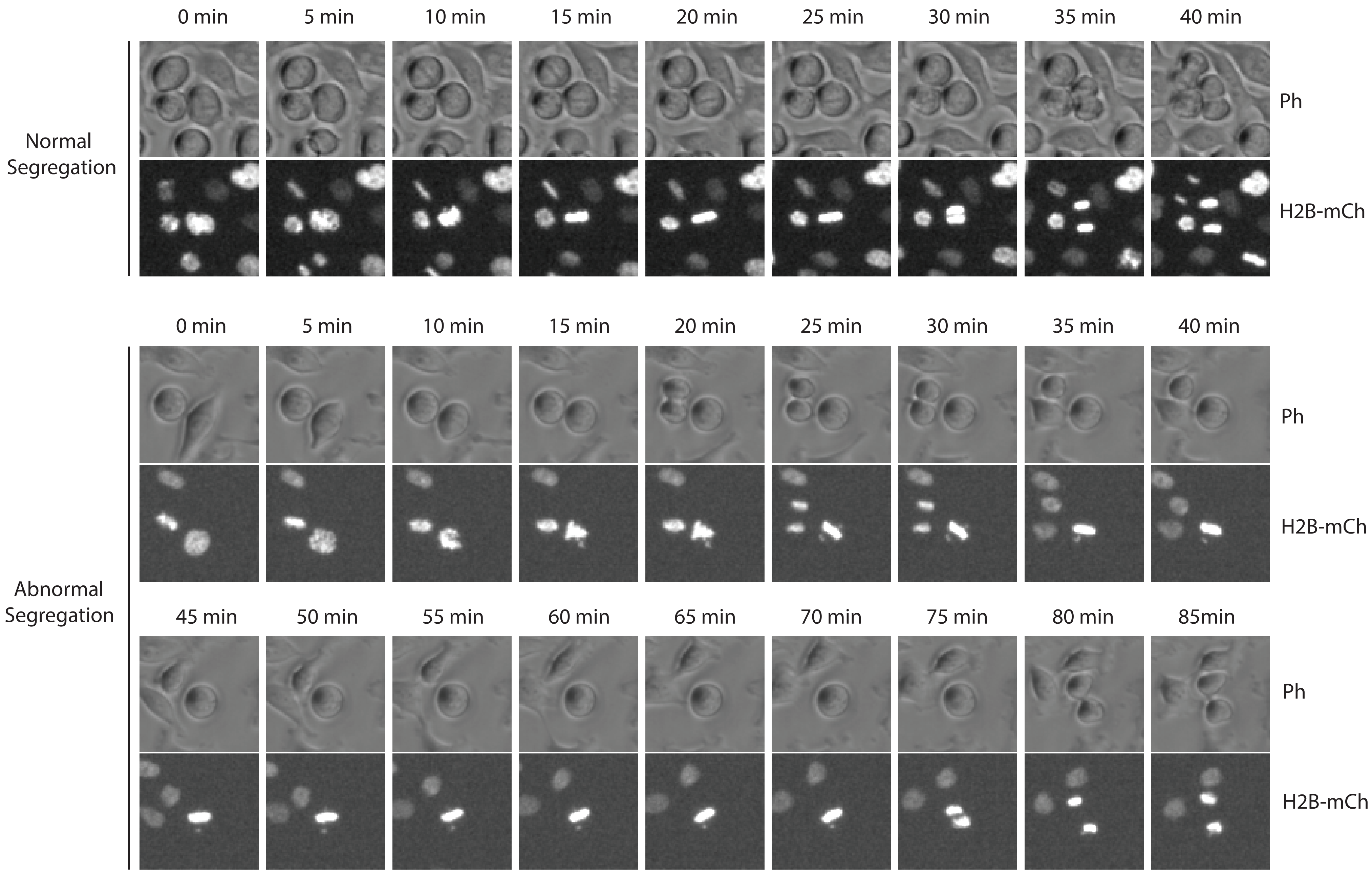

Sup Figure 4. Chromosome segregation errors after H2A.Z RNAi. HeLa Tet-on cells were first transfected by siRNA oligos targeting H2AFV or H2AFZ for 24 hrs in 96-well imaging plates and then arrested by thymidine for 20 hrs. Cells were released into mitosis with live-cell imaging. Mitotic time was counted from NEBD (nuclear envelope breakdown) to anaphase (sister chromatid separation). Majority of cells staying longer in mitosis exhibits inefficient chromsome alignment.
