# Supplementary material for: Role of noncanonical histone H2A variant, H2A.Z, to maintain proper centromeric transcription and chromosome segregation
Source: J Biol Chem. 2025 Mar 28;301(5):108464. doi: 10.1016/j.jbc.2025.108464 (PMC12051535; doi:10.1016/j.jbc.2025.108464)
Supplement: Sup Figure 5 [file mmc5.pdf]

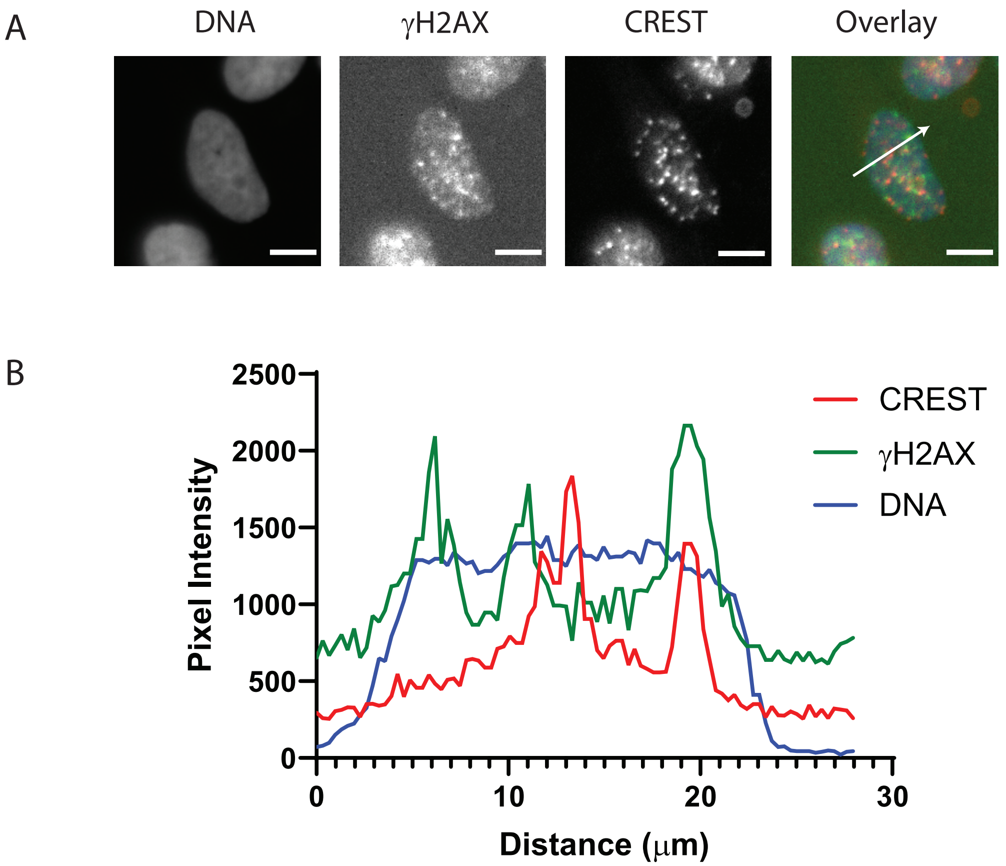

Sup Figure 5. Colocalization of CREST and γH2AX after H2A.FV RNAi. (A) Representative IF image. HeLa Tet-on cells were transfected by siRNA oligos targeting H2AFV for 48 hrs in 96-well imaging plates. Cells were fixed and stained by phospho-specific antibody against H2A.XpS139 (γH2AX) and Hoechst 33342. Bar: 10μm. (B) Pixel intensity of the arrow region in IF image. Pixel intensity of CREST (red), γH2AX (green), and DNA (blue) was shown together from left to right of the arrow. Some of CREST colocalized with γH2AX.
