# Supplementary material for: Role of noncanonical histone H2A variant, H2A.Z, to maintain proper centromeric transcription and chromosome segregation
Source: J Biol Chem. 2025 Mar 28;301(5):108464. doi: 10.1016/j.jbc.2025.108464 (PMC12051535; doi:10.1016/j.jbc.2025.108464)
Supplement: Sup Figure 6 [file mmc6.pdf]

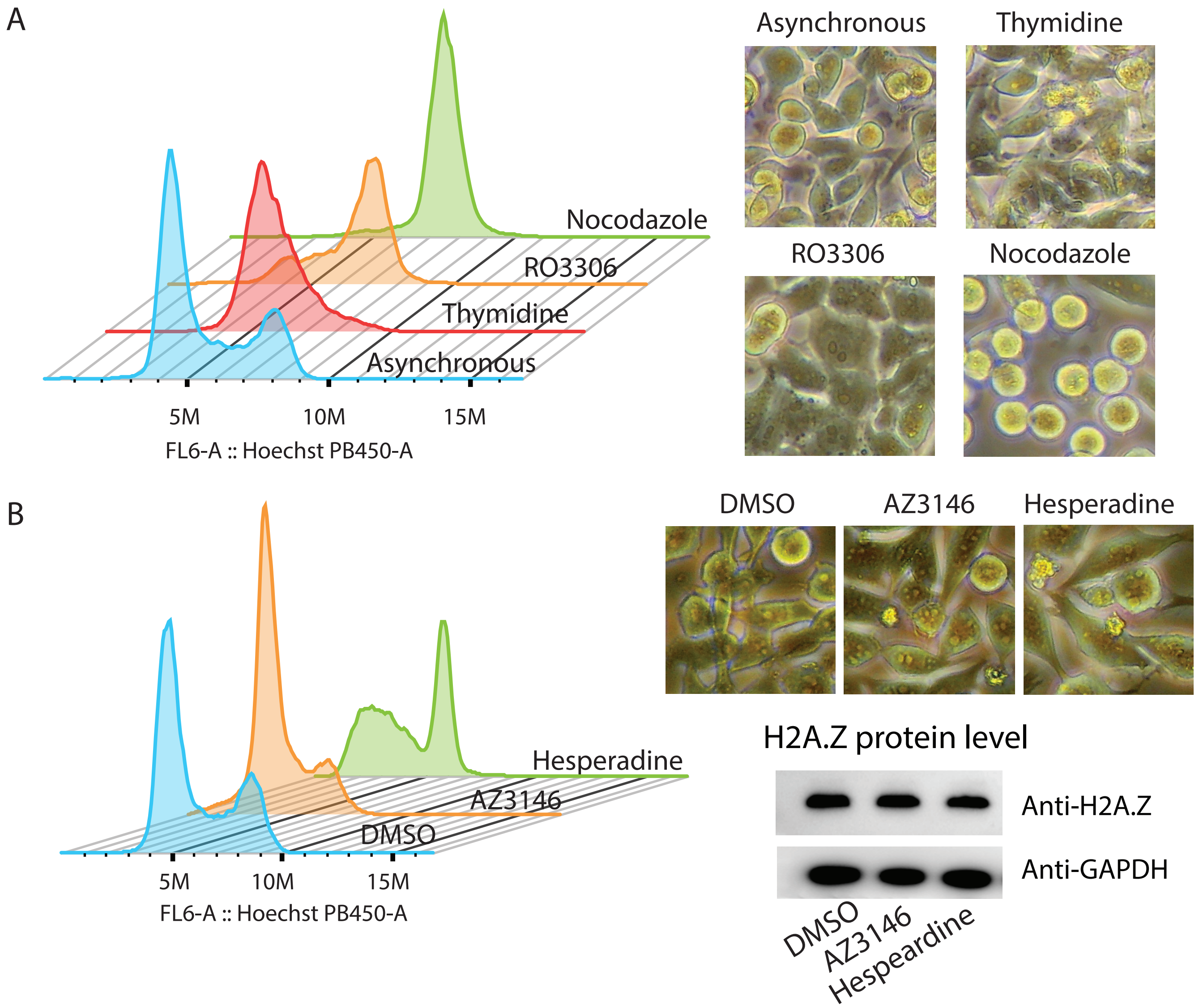

Sup Figure 6. Cell cycle profile of drug-treated cells. (A) FACS analysis of arrested cells. HeLa Tet-on cells were incubated with DMSO (asynchronous), thymidine (G1 arrest), RO3306 (G2 arrest), or nocodazole (M arrest) for 16 hrs. Live cells were imaged by microscope. Cells were then fixed, stained by Hoechst 33342, and processed for FACS. Representative phase contrast images of arrested cells were shown. (B) FACS & WB analysis of drug-treated cells. HeLa Tet-on cells were treated with AZ3146 or hesperadine for 24 hrs. Live cells were imaged by microscope. One set of cells were prepared for SDS-PAGE and WB with appropriate antibodies. The other set of cells were then fixed, stained by Hoechst 33342, and processed for FACS. Representative phase contrast images of drug-treated cells were shown.
