# Supplementary material for: Role of noncanonical histone H2A variant, H2A.Z, to maintain proper centromeric transcription and chromosome segregation
Source: J Biol Chem. 2025 Mar 28;301(5):108464. doi: 10.1016/j.jbc.2025.108464 (PMC12051535; doi:10.1016/j.jbc.2025.108464)
Supplement: Sup Figure 7 [file mmc7.pdf]

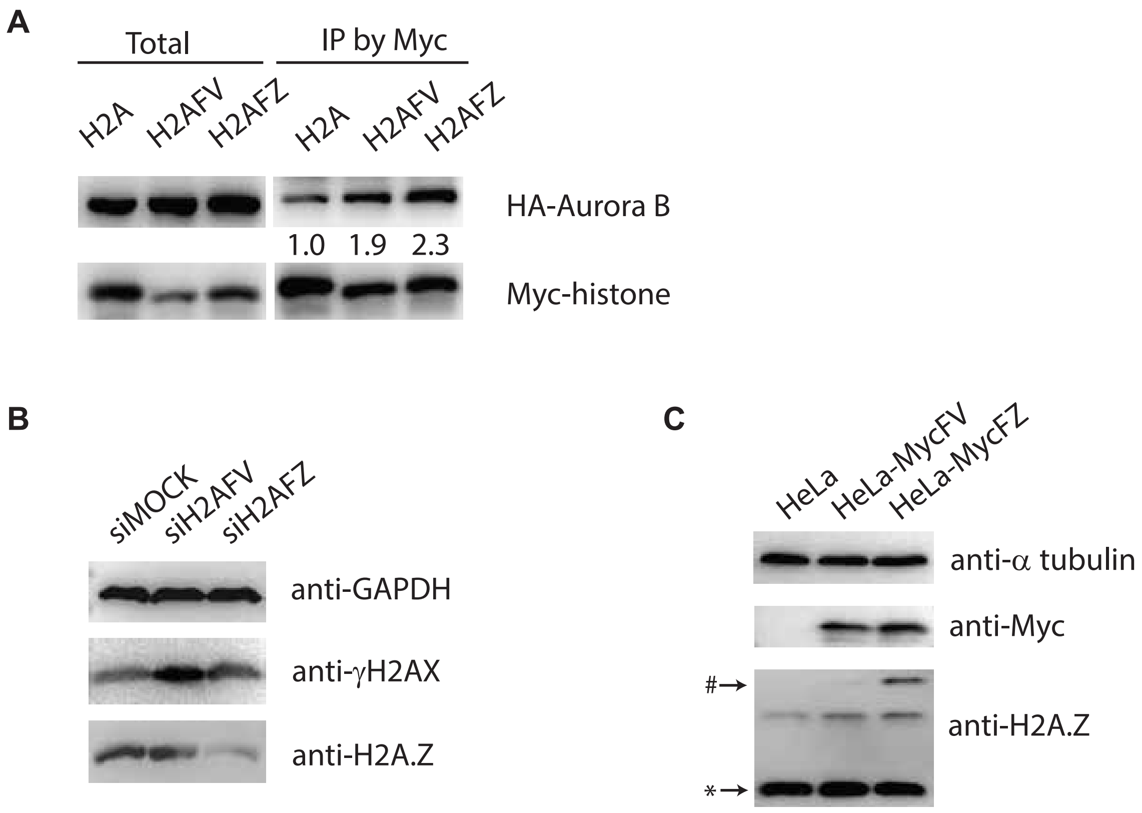

Sup Figure 7. WB results of protein interaction, DNA damages, and Myc-H2A.Z stable line. (A) CoIP experiment of Aurora B with histones. HA-Aurora B was cotransfected with Myc-H2A, Myc-H2AFV, or Myc-H2AFZ, into HeLa Tet-on cells. Cells were then arrested at M-phase with nocodazole for 16 hrs. Myc-H2AFV was pulled down by Myc beads. Total lysates and immunoprecipitated fractions were resolved by SDS-PAGE and processed for western blot. Normalization of coimmunoprecipitated signals by relevant immunoprecipitated Myc-histone signals was carried out by ImageJ gel quantitation method and the result was shown. (B) DNA damages of RNAi cells. HeLa Tet-on cells were transfected by siRNA oligos targeting H2AFV or H2AFZ for 48 hrs and total lysates were resolved by SDS-PAGE and processed for WB using appropriate antibodies. (C) Myc-H2A.Z stable lines. Myc-H2AFV or Myc-H2AFZ stable lines were cultured with doxycycline for 24 hrs and total lysates were processed by SDS-PAGE and WB. # indicates Myc-H2AFV or FZ. * indicates endogenous H2AFV or FZ.
