# Supplementary material for: Role of noncanonical histone H2A variant, H2A.Z, to maintain proper centromeric transcription and chromosome segregation
Source: J Biol Chem. 2025 Mar 28;301(5):108464. doi: 10.1016/j.jbc.2025.108464 (PMC12051535; doi:10.1016/j.jbc.2025.108464)
Supplement: Sup Figure 8 [file mmc8.pdf]

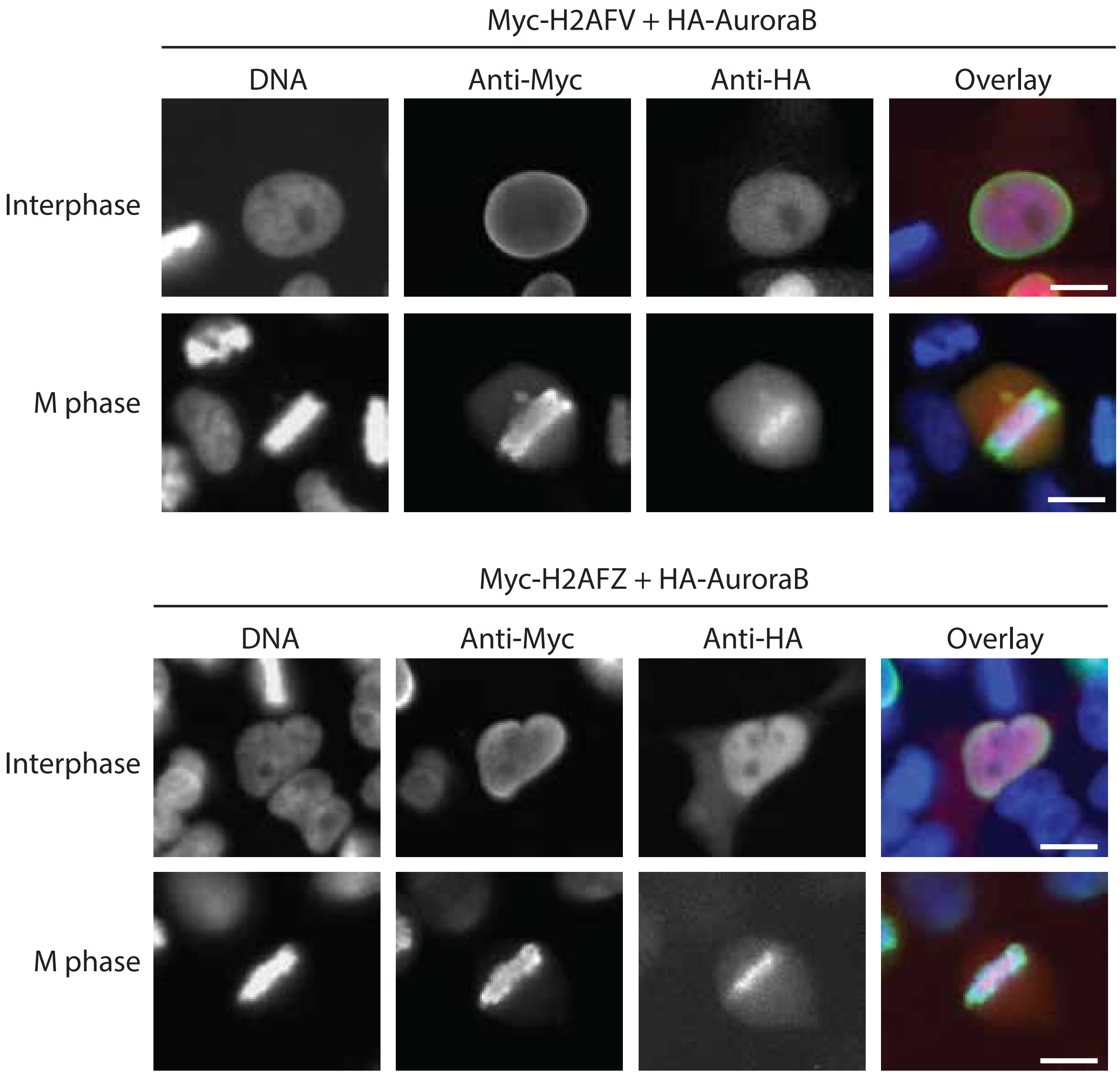

Sup Figure 8. Cellular localization of H2A.Z isoforms and Aurora B. HeLa Tet-on cells were cotransfected with Myc-H2AFV and HA-Aurora B (top) or Myc-H2AFZ and HA-Aurora B (bottom). Cells were then arrested at G2 by RO3306 treatment overnight and released into M-phase with MG132 for 1 hr. Cells were next processed for immunofluorescence with Myc and HA antibody. Representative images of their localization were shown. Size bar, 10 µM.
