# Supplementary material for: Role of noncanonical histone H2A variant, H2A.Z, to maintain proper centromeric transcription and chromosome segregation
Source: J Biol Chem. 2025 Mar 28;301(5):108464. doi: 10.1016/j.jbc.2025.108464 (PMC12051535; doi:10.1016/j.jbc.2025.108464)
Supplement: Sup Figure 9 [file mmc9.pdf]

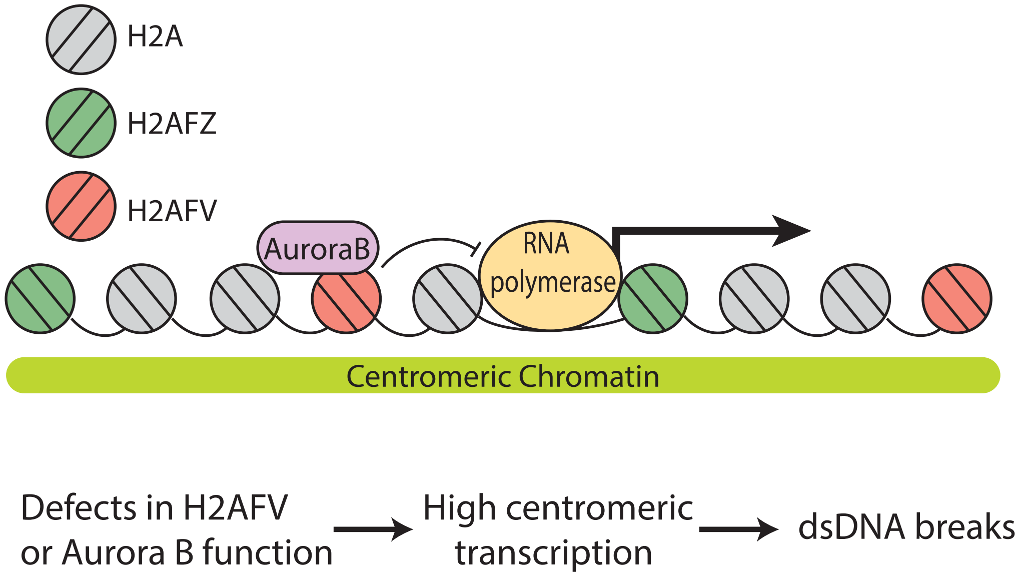

Sup Figure 9. Illustration of regulatory mechanism of centromeric transcription by H2AFV. Two isoforms of H2A.Z localizes to the centromeric chromatin and regulate proper centromeric transcription. When H2AFV function is inhibited, centromeric transcription is enhanced, which results in increased double strand DNA break due to secondary structure of cenromeric DNA. Aurora B may bind to H2AFV histone and faciliates its epigenetic modifications for its proper function. Aurora B was shown to phopsphrylate H3S10 or H3S28 for proper chromatin function. In addition, Aurora B regulates H2AFV function in centromeric transcruption.
